# *In vitro* activity of cefepime/taniborbactam and comparator agents against Gram-negative bacterial bloodstream pathogens recovered from patients with cancer

**DOI:** 10.1093/jacamr/dlae060

**Published:** 2024-04-10

**Authors:** Bahgat Gerges, Joel Rosenblatt, Y-Lan Truong, Ying Jiang, Samuel A Shelburne, Anne-Marie Chaftari, Ray Hachem, Issam Raad

**Affiliations:** Department of Infectious Diseases, Infection Control and Employee Health Research, The University of Texas MD Anderson Cancer Center, 1515 Holcombe Blvd, Houston, TX 77030, USA; Department of Infectious Diseases, Infection Control and Employee Health Research, The University of Texas MD Anderson Cancer Center, 1515 Holcombe Blvd, Houston, TX 77030, USA; Department of Infectious Diseases, Infection Control and Employee Health Research, The University of Texas MD Anderson Cancer Center, 1515 Holcombe Blvd, Houston, TX 77030, USA; Department of Infectious Diseases, Infection Control and Employee Health Research, The University of Texas MD Anderson Cancer Center, 1515 Holcombe Blvd, Houston, TX 77030, USA; Department of Infectious Diseases, Infection Control and Employee Health Research, The University of Texas MD Anderson Cancer Center, 1515 Holcombe Blvd, Houston, TX 77030, USA; Department of Genomic Medicine, The University of Texas MD Anderson Cancer Center, 1515 Holcombe Blvd, Houston, TX 77030, USA; Department of Infectious Diseases, Infection Control and Employee Health Research, The University of Texas MD Anderson Cancer Center, 1515 Holcombe Blvd, Houston, TX 77030, USA; Department of Infectious Diseases, Infection Control and Employee Health Research, The University of Texas MD Anderson Cancer Center, 1515 Holcombe Blvd, Houston, TX 77030, USA; Department of Infectious Diseases, Infection Control and Employee Health Research, The University of Texas MD Anderson Cancer Center, 1515 Holcombe Blvd, Houston, TX 77030, USA

## Abstract

**Background:**

Taniborbactam is a β-lactamase inhibitor that, when combined with cefepime, may offer a potential treatment option for patients with serious and resistant Gram-negative bacterial (GNB) pathogens.

**Objectives:**

This study evaluated *in vitro* activity of cefepime/taniborbactam and comparator agents against GNB pathogens isolated from patients with cancer at our institution.

**Methods:**

A total of 270 GNB pathogens (2019–23) isolated from patients with cancer were tested against cefepime/taniborbactam and comparator agents commonly used for these patients. CLSI-approved broth microdilution methods were used. MIC_50_, MIC_90_, MIC range and percentage of susceptibility calculations were made using FDA breakpoints when available.

**Results:**

Cefepime/taniborbactam showed highly potent activity against tested Enterobacterales, including isolates producing ESBLs and carbapenem-resistant Enterobacterales. At a provisional breakpoint of ≤16/4 mg/L, cefepime/taniborbactam inhibited most tested species of GNB pathogens, with overall 98.9% susceptibility, which was significantly (*P *< 0.0001) higher than the susceptibility of the GNB isolates to all other tested comparator agents, ranging from 39.6% for cefepime to 86.3% for ceftazidime/avibactam.

**Conclusions:**

Our results showed that taniborbactam in combination with cefepime improved *in vitro* activity against GNB pathogens isolated from patients with cancer, including MDR *Pseudomonas aeruginosa*, carbapenem-resistant Enterobacterales, ESBL-producing Enterobacterales and *Stenotrophomonas maltophilia* isolates, with highest activity compared with all tested comparator agents, including other β-lactam/β-lactamase inhibitor combinations. Further studies are warranted to explore the efficacy of cefepime/taniborbactam for empirical initial treatment of GNB infections in cancer patients with high rates of febrile neutropenia requiring hospitalization.

## Introduction

Patients with cancer are prone to develop frequent bacterial infections, especially during chemotherapy-induced neutropenia. These infections are considered oncologic emergencies, and the accepted standard of care in patients with febrile neutropenia is prompt, empirical, broad-spectrum antimicrobial therapy.^1^ The prevalence of infections due to Gram-negative bacteria (GNB) is increasing at many cancer centres across the USA, and in some centres, most infections are GNB infections.^2,3^ Cancer centres are also reporting an increasing number of infections attributed to MDR GNB.^2,3^ In a study comprising 231 facilities, the incidence of GNB with antimicrobial resistance was 40%–85% higher in cancer patients than patients without cancer.^4^ Global antibiotic resistance has reached critical levels; major bacterial pathogens have rapidly evolved to be resistant to multiple drugs, and newer agents with activity against GNB, especially resistant strains, are urgently needed.^5^

β-Lactam antibiotics, including penicillins, cephalosporins, monobactams and carbapenems, account for more than half of all antibiotic prescriptions.^6^ However, overuse of β-lactam antibiotics has resulted in the emergence and spread of β-lactam-resistant bacteria.^7^ β-Lactamases comprise four classes: A, B, C and D.^8^ Classes A, C and D include serine β-lactamases, and class B enzymes are zinc-coordinated MBLs, which have a broad substrate profile with strong carbapenemase activity.^9^

Cefepime is a fourth-generation cephalosporin β-lactam antibiotic that was approved by the US FDA in 1999 and is often used to treat severe nosocomial pneumonia and infections caused by MDR microorganisms such as *Pseudomonas aeruginosa*. Cefepime is also used for empirical treatment of febrile neutropenia,^10,11^ but cefepime resistance is often detected in blood culture isolates from these patients.^10^ One strategy for overcoming β-lactamase-mediated resistance is the coadministration of β-lactam antibiotics with an appropriate β-lactamase inhibitor. With this combination, the β-lactamase inhibitor forms a covalent adduct with the active β-lactamase enzyme, thereby preventing the enzyme from hydrolysing the β-lactam antibiotic.^12^

Taniborbactam is a novel cyclic boronate, broad-spectrum, serine β-lactamase and MBL inhibitor under development for the treatment of MDR and carbapenem-resistant bacterial infections.^13,14^ The combination of cefepime and taniborbactam may be useful for the treatment of infections in patients with cancer, but so far its activity against bacterial infections in cancer patients has not been tested.^15^ In the current study, we evaluated the *in vitro* activity of cefepime/taniborbactam against recent clinical GNB pathogens recovered from patients with cancer and compared the activity of cefepime/taniborbactam with that of other antimicrobial agents commonly used to treat GNB infections in patients with cancer.

## Materials and methods

We evaluated the *in vitro* activity of cefepime/taniborbactam, and seven comparator agents commonly used to treat GNB infections in cancer patients. Activity was tested against 270 GNB pathogens isolated recently (2019–23) from patients being treated at The University of Texas MD Anderson Cancer Center. These bacteria were isolated exclusively from blood cultures, which were processed in our institution’s clinical microbiology laboratory, stored in our Institutional Review Board-approved research repository (PA13-0334), and revived for testing as needed. The identity of each isolate was determined by MALDI-TOF. Only one isolate per patient was tested (i.e. no duplicate or serial isolates). The selection of these de-identified isolates was carried out in sequence, starting with the most recently collected specimens, to meet predetermined quotas, as agreed upon between MD Anderson and the sponsor, for each species based on their susceptibility traits. This selection process was conducted without any deliberate bias.

Taniborbactam powder was provided by the sponsor, Venatorx Pharmaceuticals Inc. (Malvern, PA, USA). Comparator agents were purchased from reliable commercial sources. Comparator agents included amikacin (Supelco, Sigma–Aldrich Lot # LRAC9136), cefepime (Supelco, Sigma–Aldrich Lot # LRAB8503), ceftazidime/avibactam, ceftazidime (Supelco, Sigma–Aldrich Lot # LRAC1680), avibactam (MedChemExpress Lot # HY-14879A), ceftolozane/tazobactam, ceftolozane (Sigma–Aldrich Batch # 0000214933) and tazobactam (Supelco, Sigma–Aldrich Lot # LRAD4754), levofloxacin (Sigma–Aldrich Batch # 0000229977), meropenem (Alfa Assar, Sigma Aldrich lot# P06E020) and piperacillin/tazobactam, piperacillin (Supelco, Sigma–Aldrich Lot # LRAD2998).

Susceptibility testing was performed using CLSI broth microdilution methods.^16,17^ Appropriate ATCC quality control organisms (*Escherichia coli* ATCC 25922, *P*. *aeruginosa* ATCC 27853 and *Klebsiella pneumoniae* ATCC-700603) were included in each run. *E. coli* NCTC 13353 was used as the routine quality control organism for confirming the activity of cefepime and the cefepime/taniborbactam combination.^16,17^

MIC_50_, MIC_90_, MIC ranges and susceptibility rates (using FDA breakpoints) were calculated according to CLSI 2022.^17^ The provisional cefepime/taniborbactam susceptibility breakpoint for GNB is ≤16/4 mg/L.^18^ Taniborbactam, avibactam and tazobactam were used at fixed concentrations of 4.0 mg/L, as recommended by CLSI.^16,17^ Statistical differences in susceptibility rates (%) of GNB pathogens to cefepime/taniborbactam and comparator agents were compared using the Fisher exact test (*P* ≤ 0.05 was considered statistically significant).

## Results

**Figure 1.**
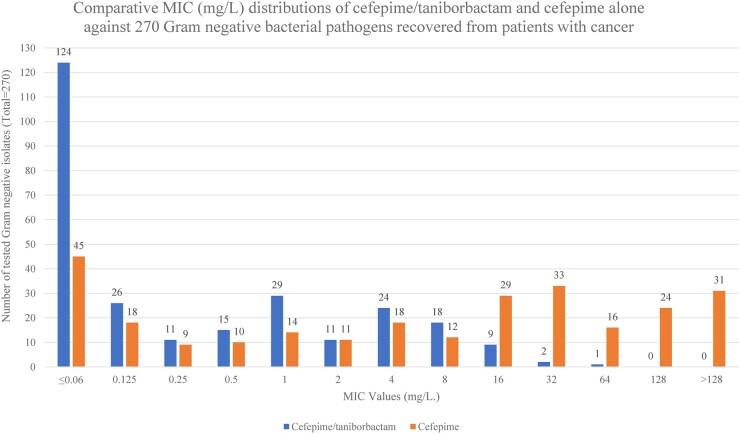
The comparative MIC distributions of cefepime/taniborbactam and cefepime alone against 270 GNB pathogens recovered from patients with cancer.

The MIC_50_ and MIC_90_ values for cefepime/taniborbactam and comparator agents against GNB pathogens are shown in Table 1, and the comparative susceptibility of GNB pathogens, including highly resistant isolates, to cefepime/taniborbactam and comparator agents is shown in Table 2. The susceptibility patterns of MDR *P. aeruginosa* and carbapenem-resistant Enterobacterales (CRE) isolates are shown in Tables 3 and 4, respectively, while the comparative MIC (mg/L) distribution of cefepime/taniborbactam in combination and cefepime alone against 270 GNB pathogens is shown in Figure 1.

**Table 1.** MIC_50_ and MIC_90_ (mg/L) of cefepime/taniborbactam and comparators against GNB isolated from patients with cancer

Species (no. of isolates)	MIC_90_ (MIC_50_)							
	FTB	FEP	CZA	C/T	MEM	TZP	LVX	AMK
*Citrobacter* spp. (*n* = 10)	0.25 (≤0.06)	4 (0.5)	0.5 (0.25)	8 (8)	≤0.06 (≤0.06)	>128 (>128)	1 (≤0.06)	2 (0.5)
*E. cloacae* (*n* = 20)	0.5 (≤0.06)	>128 (0.25)	0.5 (0.25)	8 (1)	≤0.06 (≤0.06)	>128 (64)	2 (≤0.06)	1 (1)
*E. coli* (cumulative *n = *50)	8 (≤0.06)	>128 (32)	8 (0.125)	8 (0.25)	8 (≤0.06)	>128 (16)	16 (8)	16 (2)
ESBL-producing *E. coli* (*n* = 20)	0.25 (≤0.06)	128 (64)	0.5 (≤0.06)	8 (0.25)	≤0.06 (≤0.06)	>128 (32)	16 (8)	8 (2)
Non-ESBL-producing *E. coli* (*n* = 20)	0.125 (≤0.06)	32 (0.125)	0.5 (≤0.06)	0.5 (0.125)	≤0.06 (≤0.06)	32 (8)	16 (1)	4 (2)
CRE *E. coli* (*n* = 10)	32 (8)	>128 (>128)	>128 (8)	32 (4)	64 (8)	>128 (>128)	32 (8)	>128 (16)
*K. aerogenes* (*n* = 20)	0.125 (≤0.06)	1 (≤0.06)	1 (0.25)	8 (0.25)	≤0.06 (≤0.06)	>128 (16)	0.125 (≤0.06)	2 (1)
*K. oxytoca* (*n* = 20)	0.125 (≤0.06)	32 (0.5)	1 (0.25)	1 (0.25)	≤0.06 (≤0.06)	>128 (16)	32 (≤0.06)	2 (1)
*K. pneumoniae* (cumulative *n = *70)	2 (≤ 0.06)	>128 (16)	4 (0.5)	64 (4)	16 (≤ 0.06)	>128 (64)	32 (2)	8 (2)
ESBL-producing *K. pneumoniae* (*n* = 25)	0.25 (≤0.06)	>128 (64)	1 (0.5)	64 (4)	0.125 (≤0.06)	>128 (64)	8 (2)	8 (2)
Non-ESBL-producing *K. pneumoniae* (*n* = 25)	0.125 (≤0.06)	16 (0.125)	1 (0.25)	1 (0.25)	≤0.06 (≤0.06)	128 (16)	4 (0.25)	4 (1)
CRE *K. pneumoniae* (*n* = 20)	8 (2)	>128 (>128)	16 (1)	64 (16)	>128 (4)	>128 (>128)	>32 (16)	32 (4)
*P. aeruginosa* (cumulative *n* = 40)	16 (4)	32 (16)	32 (4)	8 (2)	32 (4)	>128 (32)	16 (4)	64 (4)
MDR *P. aeruginosa* (*n* = 20)	16 (8)	64 (16)	64 (8)	16 (2)	>128 (16)	>128 (128)	32 (8)	64 (8)
Non-MDR *P. aeruginosa* (*n* = 20)	4 (1)	16 (2)	8 (1)	4 (1)	16 (0.125)	>128 (16)	4 (0.25)	4 (2)
*S. marcescens*	1 (≤0.06)	1 (0.125)	0.5 (0.5)	2 (0.5)	0.125 (≤0.06)	32 (8)	0.125 (≤0.06)	4 (2)
*S. maltophilia* (*n* = 30)	8 (4)	64 (32)	128 (32)	>128 (32)	>128 (128)	>128 (>128)	8 (0.5)	>128 (32)

FTB, cefepime/taniborbactam; FEP, cefepime; CZA, ceftazidime/avibactam; C/T, ceftolozane/tazobactam; MEM, meropenem; TZP, piperacillin/tazobactam; LVX, levofloxacin; AMK, amikacin.

**Table 2.** Susceptibility rates (%) to cefepime/taniborbactam and comparators for GNB isolates, including highly resistant pathogens, from patients with cancer

Species	No. tested isolates	No. susceptible isolates (% susceptibility)							
		FTB	FEP	CZA	C/T	MEM	TZP	LVX	AMK
ESBL-producing *E. coli*	20	20 (100)	1 (5)	20 (100)	17 (85)	20 (100)	9 (45)	2 (10)	20 (100)
CRE *E. coli*	10	8 (80)	0 (0)	5 (50)	2 (20)	3 (30)	1 (10)	0 (0)	6 (60)
ESBL-producing *K. pneumoniae*	25	25 (100)	2 (8)	25 (100)	10 (40)	25 (100)	7 (28)	8 (32)	25 (100)
CRE *K. pneumoniae*	20	20 (100)	0 (0)	16 (80)	0 (0)	3 (15)	3 (15)	0 (0)	15 (75)
MDR *P. aeruginosa*	20	19 (95)	0 (0)	14 (70)	13 (65)	2 (10)	5 (25)	0 (0)	13 (65)
*S. maltophilia*	30	30 (100)	0 (0)	10 (33.3)	4 (13.3)	0 (0)	0 (0)	17 (56.7)	8 (26.7)
Total resistant isolates	125	122 (97.6)	3 (2.4)	90 (72.0)	46 (36.8)	53 (42.4)	25 (20.0)	27 (21.6)	87 (69.6)
Total non-resistant isolates	145	145 (100.0)	104 (71.7)	143 (98.6)	122 (84.1)	136 (93.8)	82 (56.6)	111 (76.6)	144 (99.3)
Total, all tested isolates	270	267 (98.9)	107 (39.6)	233 (86.3)	168 (62.2)	191 (70.7)	107 (39.6)	138 (51.1)	231 (85.6)

FTB, cefepime/taniborbactam; FEP, cefepime; CZA, ceftazidime/avibactam; C/T, ceftolozane/tazobactam; MEM, meropenem; TZP, piperacillin/tazobactam; LVX, levofloxacin; AMK, amikacin.

**Table 3.** The susceptibility patterns of 20 MDR *P. aeruginosa* isolates versus all tested antimicrobial agents

Agents	Susceptibility pattern of 20 MDR *P. aeruginosa* isolates (mg/L)												
	<0.06	0.125	0.25	0.5	1	2	4	8	16	32	64	128	>128
FTB	0	0	1	0	1	1	3	8	5	0	1	0	0
FEP	0	0	0	0	0	0	1	3	7	6	1	1	1
CZA	0	0	0	0	2	2	5	5	1	1	2	1	1
C/T	0	0	0	0	1	12	2	2	1	0	1	1	0
MEM	0	0	1	1	0	0	1	3	6	5	0	0	3
TZP	0	0	0	0	0	0	1	1	3	0	2	3	10
LVX	0	0	0	0	2	0	5	4	5	2	>32: (2)^a^		
AMK	0	0	0	0	0	1	5	4	3	2	3	1	1

FTB, cefepime/taniborbactam; AMK, amikacin; FEP, cefepime; CZA, ceftazidime/avibactam; C/T, ceftolozane/tazobactam; MEM, meropenem; TZP, piperacillin/tazobactam; LVX, levofloxacin.

^a^The concentrations of all tested antimicrobial agents ranged from 0.06 to 128 mg/L except LVX (from 0.06 to 32 mg/L).

**Table 4.** The susceptibility patterns of 30 CRE (10 *E.coli* + 20 *K. pneumoniae*) isolates versus all tested antimicrobial agents

Agents	Susceptibility pattern of 30 CRE isolates; 10 *E. coli* + (20 *K. pneumoniae*) (mg/L)												
	<0.06	0.125	0.25	0.5	1	2	4	8	16	32	64	128	>128
FTB	0	0 + (1)	0	1 + (2)	1 + (5)	1 + (5)	0 + (3)	3 + (3)	2 + (1)	2 + (0)	0	0	0
FEP	0	0	0	0	0	0	0 + (1)	0 + (1)	0 + (1)	0 + (1)	0	3 + (3)	7 + (13)
CZA	0	0	0 + (2)	1 + (2)	0 + (6)	2 + (2)	0 + (2)	2 + (2)	0 + (2)	0	0 + (1)	0	5 + (1)
C/T	0	0	0	0	2 + (0)	0	3 + (1)	1 + (1)	2 + (8)	1 + (5)	1 + (3)	0 + (1)	0 + (1)
MEM	0	0	0	0	3 + (3)	0 + (4)	0 + (3)	3 + (1)	1 + (2)	1 + (1)	1 + (1)	0 + (1)	1 + (4)
TZP	0	0	0	0	0	0	0	0 + (2)	1 + (1)	0	1 + (0)	0	8 + (17)
LVX	0	0	0	0	0 + (1)	0	2 + (0)	3 + (2)	3 + (7)	1 + (5)	>32: 1+ (5)^a^		
AMK	0	0	0 + (1)	0 + (2)	0 + (3)	1 + (2)	1 + (5)	1 + (0)	3 + (2)	0 + (3)	1 + (0)	0	3 + (2)

FTB, cefepime/taniborbactam; AMK, amikacin; FEP, cefepime; CZA, ceftazidime/avibactam; C/T, ceftolozane/tazobactam; MEM, meropenem; TZP, piperacillin/tazobactam; LVX, levofloxacin.

^a^The concentrations of all tested antimicrobial agents ranged from 0.06 to 128 mg/L, except LVX (from 0.06 to 32 mg/L).

Cefepime/taniborbactam at a tentative susceptible breakpoint of ≤16/4 mg/L inhibited 100% of all tested isolates of *Citrobacter* species, *Enterobacter cloacae*, non-carbapenem-resistant *E. coli* including ESBL-producing isolates, *Klebsiella aerogenes*, *Klebsiella oxytoca*, *K*. *pneumoniae* including both ESBL- and CRE-producing isolates, non-MDR *P*. *aeruginosa, Serratia marcescens* and *Stenotrophomonas maltophilia*. Cefepime/taniborbactam inhibited 80% of CRE *E. coli* isolates and 95% of MDR *P*. *aeruginosa* isolates.

Susceptibility rates of the GNB isolates to cefepime/taniborbactam were significantly higher than for all other tested comparator agents (*P* < 0.0001), as shown in Table 1.

The comparative MIC distributions of cefepime/taniborbactam and cefepime alone against 270 GNB pathogens showed that 124 (45.9%) and 45 (16.7%) of the tested isolates were susceptible to cefepime/taniborbactam and cefepime alone at ≤0.06 mg/L, respectively, while 3 (1.1%) and 104 (38.5%) of the tested isolates were resistant to cefepime/taniborbactam and cefepime alone at ≥ 16 mg/L, respectively.

Cefepime/taniborbactam was significantly more active than cefepime, piperacillin/tazobactam and levofloxacin against non-carbapenem-resistant ESBL-producing *E. coli* in terms of (provisional) susceptibility rates (*P* < 0.0001), but cefepime/taniborbactam was not significantly different from ceftolozane/tazobactam against ESBL-producing *E. coli*. Cefepime/taniborbactam was also significantly more active *in vitro* than cefepime (*P* < 0.001), ceftolozane/tazobactam (*P* = 0.023), piperacillin/tazobactam (*P* = 0.006) and levofloxacin (*P* < 0.001) against CRE *E.coli*, but cefepime/taniborbactam was not significantly different from ceftazidime/avibactam, meropenem or amikacin against CRE *E. coli*. Cefepime/taniborbactam had significantly improved *in vitro* activity compared with cefepime, ceftolozane/tazobactam, piperacillin/tazobactam and levofloxacin (*P* < 0.0001) against ESBL-producing *K*. *pneumoniae* and compared with cefepime, ceftolozane/tazobactam, meropenem, piperacillin/tazobactam, levofloxacin (*P* < 0.0001) and amikacin (*P* = 0.047) against CRE *K*. *pneumoniae*. Cefepime/taniborbactam activity was not significantly different from that of ceftazidime/avibactam against CRE *K*. *pneumoniae*.

## Discussion

Our data showed that cefepime/taniborbactam had excellent antibacterial activity against most isolates of tested GNB pathogens that recently caused serious infections in patients with cancer, including ESBL, CRE and MDR isolates. We found that addition of taniborbactam to cefepime rendered resistant ESBL-producing Enterobacterales, CRE and MDR *P*. *aeruginosa* isolates susceptible to cefepime. Cefepime alone showed activity against only 39.6% of the 270 tested GNB isolates, whereas cefepime/taniborbactam showed activity against 98.9% of isolates. Cefepime alone showed activity against only 2.4% of 125 resistant isolates (ESBL, CRE and MDR isolates and *S. maltophilia*), whereas cefepime/taniborbactam showed activity against 97.6% of the same isolates, and these differences were highly significant (*P* < 0.0001). These results are consistent with the results of other investigations of cefepime/taniborbactam that did not specifically focus on isolates from patients with cancer.^19,20^

Bacterial resistance to antimicrobial agents is increasing, with increasing rates of infections caused by ESBL, CRE and MDR pathogens.^21,22^ Treatment options for infections caused by these resistant pathogens are limited and often associated with high rates of clinical failure.^23,24^ In addition, GNB pathogens present in immunosuppressed patients with cancer tend to be more resistant because these patients are usually exposed to prolonged prophylactic and empirical antimicrobial therapy. When we tested cefepime/taniborbactam and several currently used antibiotics to treat infections with resistant GNB pathogens, we showed that cefepime/taniborbactam had significantly (*P* < 0.0001) improved *in vitro* activity against resistant isolates from patients with cancer. Only 3 of the 270 tested GNB isolates (1.1%; including 2 CRE *E. coli* isolates and 1 MDR *P. aeruginosa* isolate) were resistant to cefepime/taniborbactam, whereas 60.4% of the tested isolates were resistant to cefepime alone and 13.7% were resistant to ceftazidime/avibactam.

Resistance to carbapenems is mediated by several mechanisms, including transferable carbapenemase enzymes. Carbapenemases are classified into different molecular classes: class A (e.g. KPC and GES); class B or MBLs (e.g. VIM, IMP, and NDM); and class D, or oxacillinases (e.g. OXA-23, −40, −58 or −48 types).^25^ Overall, carbapenemases are commonly plasmid mediated and are mainly reported in MDR Enterobacterales, *P. aeruginosa* and *Acinetobacter* species isolates.^26^ Resistance to cefepime/taniborbactam has been reported through the substitution of amino acids in the NDM enzyme and VIM enzyme.^27,28^ Interestingly, two of the three isolates resistant to cefepime/taniborbactam in this study were gene sequenced; one isolate (*E. coli*) carried the CTX-M-15/NDM-5 genes^29^ and the other (*P. aeruginosa*) had the NDM-1 gene (unpublished data). We hypothesize that these NDM MBL genes may be conferring the resistance of these isolates to cefepime/taniborbactam.

*In vitro* activity of cefepime/taniborbactam against MDR *P. aeruginosa* isolates (95%) exceeded that of ceftazidime/avibactam (70%), ceftolozane/tazobactam (65%), amikacin (65%), piperacillin/tazobactam (25%), meropenem (10%), levofloxacin (0%) and cefepime (0%). These results are consistent with those of Hernández-García *et al*.,^30^ who studied the *in vitro* activity of cefepime/taniborbactam against CRE and *P. aeruginosa* isolates recovered in Spain. That study showed that *in vitro* activity of cefepime/taniborbactam against *P. aeruginosa* isolates exceeded that of ceftazidime/avibactam, ceftolozane/tazobactam, imipenem/relebactam and meropenem/vaborbactam.^30^ The current study also showed that cefepime/taniborbactam displayed greater activity than meropenem. Meropenem showed activity against only 70.7% of isolates, whereas cefepime/taniborbactam showed activity against 98.9% (*P* < 0.0001).

Although ceftolozane/tazobactam is considered the drug of choice against *P. aeruginosa* infection, followed by ceftazidime/avibactam, our results showed that there was a chance of 30% and 35% of MDR *P. aeruginosa* isolates being resistant and not inhibited by ceftolozane/tazobactam and ceftazidime/avibactam, respectively. These findings are highly concerning as sepsis secondary to MDR *P. aeruginosa* in neutropenic patients carries a high risk of death and increases hospital length of stay and costs. This finding should definitely prompt further clinical evaluation of cefepime/taniborbactam in patients with cancer who belong to a high-risk group.

Standard-of-care guidelines pertaining to patients with high-risk febrile neutropenia recommend the use of parenteral broad-spectrum antipseudomonal β-lactam antibiotics that cover GNB pathogens as part of the initial treatment.^1^ This is based on data from a large number of studies showing that high-risk cancer patients with febrile neutropenia are likely to have a GNB infection, and if GNB are not adequately covered with an active broad-spectrum agent, these patients are likely to face grave consequences, including septic shock and possible death. In the latest IDSA guideline on the management of febrile neutropenia, published in 2011, cefepime, piperacillin/tazobactam and meropenem were listed as broad-spectrum antimicrobial agents that could be used empirically in the initial treatment of high-risk cancer patients with febrile neutropenia requiring hospitalization.^1^ However, more than a decade later, these agents do not cover the growing number of resistant GNB pathogens, such as ESBL-producing and CRE isolates. Hence, initial empirical therapy with a broad-spectrum agent such as cefepime/taniborbactam in high-risk cancer patients with febrile neutropenia requiring hospitalization needs to be studied in prospective randomized trials comparing this approach with the current standard of care, because cefepime/taniborbactam provides a broader-spectrum alternative that would cover resistant pathogens that could have a life-threatening impact on these high-risk patients.

Our study has some limitations. Although we reported MIC results, we did not perform time–kill experiments, and therefore we could not relate expected clinical activity to achievable plasma threshold. Another limitation is that this was strictly a laboratory study. The isolates collected and tested were de-identified, and hence we did not have demographic information on the patients.

In conclusion, our results showed that taniborbactam in combination with cefepime improved *in vitro* activity against GNB pathogens isolated from patients with cancer, including MDR *P. aeruginosa*, CRE, ESBL-producing Enterobacterales and *S. maltophilia* isolates with highest activity (98.9%) compared with all tested comparator agents, including other β-lactam/β-lactamase inhibitor combinations. Further studies are warranted to explore the efficacy of cefepime/taniborbactam in the empirical initial treatment of high-risk cancer patients with febrile neutropenia requiring hospitalization.
